# *Fusobacteriumnucleatum*: Pathophysiological and Clinical Involvement in Inflammatory Bowel Diseases, Colorectal Cancer and Cardiovascular Diseases

**DOI:** 10.3390/cancers17203348

**Published:** 2025-10-17

**Authors:** Vincenzo Quagliariello, Pietro Forte, Giuliana Ciappina, Luigi Colarusso, Carlotta Giorgi, Francesco Fiorica, Antonio Bottari, Giordana Di Mauro, Nicola Maurea, Massimiliano Berretta

**Affiliations:** 1 Division of Cardiology, Istituto Nazionale Tumori-IRCCS-Fondazione G. Pascale, 80131 Napoli, Italy; pietro.forte@istitutotumori.na.it (P.F.); n.maurea@istitutotumori.na.it (N.M.); 2 Section of Experimental Medicine, Department of Medical Sciences, University of Ferrara, 44121 Ferrara, Italy; giuliana.ciappina@unife.it (G.C.); carlotta.giorgi@unife.it (C.G.); mberretta@unime.it (M.B.); 3 Department of Clinical and Experimental Medicine, University of Messina, 98131 Messina, Italy; luigi.colarusso@polime.it; 4 Department of Clinical Oncology, AULSS 9 Scaligera, 37122 Verona, Italy; francesco.fiorica@aulss9.veneto.it; 5 Department of Biomedical Sciences and Morphologic and Functional Imaging, University of Messina, 98131 Messina, Italy; bottaria@unime.it; 6 Division of Medical Oncology, AOU “G. Martino” Hospital, University of Messina, 98131 Messina, Italy; giordana.dimauro@studenti.unime.it

**Keywords:** microbiota, colon cancer, cardiotoxicity, cardiovascular diseases, heart failure, drug resistance

## Abstract

*Fusobacterium nucleatum* is a Gram-negative anaerobe that occupies a central ecological niche in oral biofilms but has emerged as a trans-compartmental pathogen implicated in gastrointestinal and cardiovascular diseases. In inflammatory bowel diseases, *Fusobacterium nucleatum* adheres to the intestinal epithelium via adhesins such as FadA, disrupts tight junctions, and induces Toll-like receptor–mediated inflammatory cascades, amplifying epithelial permeability and sustaining mucosal inflammation. In colorectal cancer, *Fusobacterium nucleatum* promotes carcinogenesis through multiple mechanisms, including β-catenin activation, modulation of oncogenic microRNAs, and immune evasion via Fap2–TIGIT signaling, while also fostering a pro-inflammatory and immunosuppressive tumor microenvironment. Its enrichment correlates with advanced tumor stage, chemoresistance, and poor prognosis, underscoring its potential as a biomarker and therapeutic target. Beyond the gut, *Fusobacterium nucleatum* has been detected in atherosclerotic plaques and endocardial tissues, where it contributes to endothelial dysfunction, foam cell formation, oxidative stress, and plaque instability, thereby linking chronic oral infection with cardiovascular risk. Collectively, evidence suggests that *Fusobacterium nucleatum* acts as a pathophysiological connector across IBD, CRC, and CVD through conserved mechanisms of adhesion, immune modulation, and inflammation. Understanding these processes provides opportunities for innovative interventions, ranging from targeted antimicrobials and host-directed therapies to dietary and microbiome-based strategies, aimed at mitigating the systemic burden of this organism and improving clinical outcomes across multiple diseases.

## Simple Summary

The oral commensal *Fusobacterium nucleatum* has recently been recognized as a pathobiont with systemic implications extending well beyond periodontal disease. Increasing evidence links this bacterium to inflammatory bowel diseases, colorectal cancer, and cardiovascular disorders. Its ability to adhere to epithelial and endothelial cells, modulate the immune response, and alter local metabolic and inflammatory pathways enables it to participate in disease initiation, progression, and complications. In colorectal cancer, *Fusobacterium nucleatum* promotes tumor growth, metastasis, and drug resistance, while in inflammatory bowel diseases, it exacerbates barrier dysfunction and chronic inflammation. In cardiovascular disease, its systemic dissemination contributes to vascular inflammation, atherogenesis, and adverse cardiac remodeling. This review critically evaluates the mechanistic pathways and clinical evidence connecting *Fusobacterium nucleatum* to these major disorders and highlights potential therapeutic strategies aimed at reducing its pathogenic burden.

## 1. Introduction

*Fusobacterium nucleatum* is a Gram-negative, obligate anaerobic bacillus belonging to the *Fusobacteriaceae* family, widely recognized as a commensal of the human oral cavity and a structural keystone of dental biofilms [1]. Within the oral microbiome, it functions as a “bridging” organism that promotes the integration of early and late colonizers, sustaining complex multispecies communities through adhesion, nutrient exchange, and metabolic cross-talk [2]. Under conditions of dysbiosis, however, *Fusobacterium nucleatum* can act as a pathobiont, expanding in periodontal niches and contributing to chronic inflammation and tissue destruction [3]. Beyond its role in oral disease, accumulating evidence over the past decade has revealed a broader pathogenic potential, with strong associations documented between *F. nucleatum* and several systemic conditions [4]. One of the most striking findings has been the consistent enrichment of *Fusobacterium nucleatum* in colorectal cancer (CRC) [5]. Initial metagenomic studies demonstrated increased bacterial DNA and RNA levels in tumor tissues compared with adjacent normal mucosa, later confirmed by isolation of live organisms from patient-derived biopsies and xenograft models [6]. It is important to note, however, that most clinical and sequencing studies have focused on a limited subset of *Fusobacterium nucleatum* strains, predominantly those belonging to the nucleatum and animalis subspecies. Recent genomic data indicate that *F. nucleatum* encompasses multiple genetically distinct clades with variable virulence traits and host interactions, yet the clinical relevance of this diversity remains underexplored. The paucity of strain-level resolution in human studies represents a key limitation in interpreting associations between *F. nucleatum* and disease outcomes.

Subsequent analyses have shown that *Fusobacterium nucleatum* is not uniformly distributed across colorectal tumors but more frequently detected in right-sided cancers, suggesting site-specific ecological niches within the intestine [7]. Its higher prevalence in stool samples from CRC patients compared to healthy controls further supports its potential as a biomarker. Importantly, abundance correlates with tumor stage, metastatic spread, and unfavorable prognosis, indicating that *Fusobacterium nucleatum* may actively participate in cancer progression rather than being a passive passenger [8]. The pathogenic versatility of *F. nucleatum* is not restricted to the colorectum [9]. The bacterium has been identified in extraintestinal malignancies, including breast cancer, where it is hypothesized to reach distant tissues via hematogenous dissemination [10]. Its selective binding to tumor-associated glycans, such as Gal-GalNAc, provides a plausible mechanism for tropism toward dysplastic and neoplastic epithelia in different organ systems [11].

In this narrative review, we summarize current knowledge on *Fusobacterium nucleatum* as a mutualist, infectious agent, and *oncobacterium* [12]. We particularly focus on its pathophysiological and clinical involvement in inflammatory bowel diseases, colorectal cancer, and cardiovascular diseases, highlighting shared mechanistic pathways and their translational implications [13]. By integrating evidence from experimental, clinical, and epidemiological studies, we aim to provide a comprehensive re-evaluation of this organism as a microbial driver of inflammation, carcinogenesis, and systemic vascular pathology [14].

## 2. Methods

A narrative literature search was performed using the MEDLINE (via PubMed) and EMBASE databases to identify relevant original research articles addressing the role of *Fusobacterium nucleatum* in human disease [15]. The search covered the last 15 years (January 2010–July 2025) to capture the most updated clinical and experimental evidence. Studies were included if they met all the following criteria:

◦ Published in English with an available abstract;
◦ Reported original data from clinical studies, cohort investigations, randomized controlled trials, or quantitative meta-analyses of such studies;
◦ Addressed at least one of the following topics: *Fusobacterium nucleatum* and inflammatory bowel disease (IBD), *F. nucleatum* and colorectal cancer (CRC), *F. nucleatum* and cardiovascular diseases (CVD), microbial biomarkers, immune modulation, or microbiota–host interactions [16].

Exclusion criteria were:

◦ Narrative or systematic reviews without pooled analysis, case reports, and editorials;
◦ Studies lacking a direct investigation of *Fusobacterium nucleatum*;
◦ Experimental works unrelated to IBD, CRC, or CVD.

The search strategy incorporated Boolean operators (AND, OR) without truncation (*) to optimize retrieval of relevant articles. The exact search strings applied in each database are reported in Table 1.

The database search identified *n* = 512 records in MEDLINE and *n* = 437 in EMBASE. Specifically, the following numbers of articles were retrieved for each main string:

◦ “*Fusobacterium nucleatum* AND colorectal cancer” → *n* = 182 articles;
◦ “*Fusobacterium nucleatum* AND inflammatory bowel disease” → *n* = 64 articles;
◦ “*Fusobacterium nucleatum* AND cardiovascular disease” → *n* = 71 articles;
◦ “*Fusobacterium nucleatum* AND immune modulation/microbiota” → *n* = 98 articles.

After removal of duplicates and screening of titles and abstracts, *n* = 174 full-text articles were assessed for eligibility; of these, *n* = 136 were finally included in the review after applying the predefined exclusion criteria. Notably, we qualitatively synthesized the findings from eligible studies, integrating data from preclinical and translational research with clinical evidence to provide a comprehensive overview of the role of *F. nucleatum* in mucosal and vascular pathology [17]. The last access to the databases was on 17 July 2025 [18,19,20].

## 3. *Fusobacterium nucleatum*: An Overview of Clinical Functions and Pathogenesis

*Fusobacterium nucleatum* is a Gram-negative, spindle-shaped, obligate anaerobic bacterium that occupies a central ecological position within the human oral microbiome [21]. As a member of the *Fusobacteriaceae* family, it serves as a key “bridging” species in dental biofilms, mediating the co-aggregation of early colonizers such as Streptococcus spp. and late colonizers including *Porphyromonas gingivalis* and *Treponema denticola*, thereby facilitating the structural maturation of complex multispecies communities [22,23]. This bridging capacity is largely enabled by outer membrane adhesins such as RadD and Fap2, which promote interbacterial interactions and biofilm stability [24].

Beyond its commensal role in maintaining oral microbial ecology, *Fusobacterium nucleatum* possesses a broad virulence arsenal that allows it to shift toward pathogenicity under dysbiotic conditions. Its cell surface structures, including lipopolysaccharides, hemolysins, and proteases, can elicit robust inflammatory responses, while its ability to invade epithelial and endothelial cells enables persistence and immune evasion [25,26,27]. The bacterium’s metabolic products, such as butyrate and hydrogen sulfide, may further impair epithelial integrity and modulate immune cell function [28].

Clinically, *Fusobacterium nucleatum* has been linked to a spectrum of local and systemic conditions. Within the oral cavity, it contributes to periodontal inflammation and tissue destruction [25,26], whereas hematogenous dissemination from oral sites has been associated with extraoral infections, including adverse pregnancy outcomes, liver and brain abscesses, and Lumiere’s syndrome [29,30,31]. The detection of *Fusobacterium nucleatum* DNA within atherosclerotic plaques and other distant tissues suggests a potential role in systemic inflammatory and vascular disorders [32,33].

Moreover, its enrichment in intestinal mucosa and tumor tissues has prompted investigation into its possible contribution to gastrointestinal inflammation and carcinogenesis [34,35,36,37,38]. Although the specific mechanisms linking *Fusobacterium nucleatum* to these extraoral diseases differ, they commonly involve adhesion to host cells, activation of pro-inflammatory signaling, and modulation of the immune microenvironment [39,40,41]. This pathogenic versatility illustrates how a normally commensal organism can act as a systemic opportunist when ecological balance is disrupted [42,43,44,45,46].

Therefore, *Fusobacterium nucleatum* exemplifies the dualistic nature of the oral microbiota, serving as both an important ecological stabilizer and a potential mediator of systemic pathology. Its ability to interact with diverse host tissues underscores the clinical relevance of oral–systemic microbial connections and justifies further investigation into its pathogenic mechanisms and translational implications [47,48,49].

## 4. *Fusobacterium nucleatum* and Inflammatory Bowel Diseases: Pathogenesis and Clinical Evidence

The direct cause of inflammatory bowel disease (IBD) remains unknown; however, the gut microbiota is recognized as a crucial factor in the development of both ulcerative colitis (UC) and Crohn’s disease (CD) [50]. The complexity and heterogeneity of human gut microbiota have made it difficult to identify individual species specifically driving IBD exacerbations, although some, such as adherent-invasive *Escherichia coli* and *Fusobacterium varium*, have been proposed as relevant agents.

Increasingly, *F. nucleatum* has been isolated at significant frequency from intestinal biopsies of IBD patients, suggesting a persistent presence and a potential pro-inflammatory role [51]. Clinical and experimental studies indicate that *Fusobacterium nucleatum* may contribute to IBD pathogenesis through complementary, stage-specific mechanisms (Table 2).

Persistent colonization of the intestinal mucosa, documented by repeated isolation from biopsy material, is more frequent in IBD than in healthy controls [51,52].

Such colonization represents the first step for downstream pathogenic interactions. Invasion of the intestinal epithelium, first described in oral infections but subsequently demonstrated in the intestinal Caco-2 cell model, further compromises the epithelial barrier and promotes mucosal inflammation [53]. These processes are amplified by the organism’s ability to form biofilms, largely mediated by the outer-membrane adhesin RadD, which fosters interspecies aggregation, enhances microbial persistence, and supports colonization of mucosal surfaces [60]. Adhesion to and invasion of host cells is further strengthened by the adhesin FadA. The active pre-FadA/mFadA complex is indispensable for binding to epithelial cells, thereby facilitating mucosal colonization and promoting local inflammatory responses [57]. Notably, FadA can engage E-cadherin on intestinal epithelial cells, activating the β-catenin signaling cascade, which induces pro-inflammatory mediators and stimulates epithelial proliferation, a process that bridges chronic inflammation in IBD with the enhanced risk of CRC [58,61]. In vivo and clinical evidence link these molecular effects with disease activity. Disruption of epithelial tight-junctions by *Fusobacterium nucleatum* increases mucosal permeability, allowing translocation of luminal bacteria and antigens, which in turn drive Th17-dominated mucosal immune responses.

Recruitment of neutrophils leads to the release of neutrophil extracellular traps (NETs), further amplifying local inflammation and tissue damage. Several studies have reported positive correlations between mucosal *Fusobacterium nucleatum* burden and clinical indicators of active disease, endoscopic severity scores, fecal calprotectin concentrations, and serum C-reactive protein (CRP) levels, supporting its role as a potential biomarker of IBD activity (Table 2).

Moreover, the barrier dysfunction caused by *Fusobacterium nucleatum* may have systemic consequences. Enhanced epithelial permeability facilitates microbial and endotoxin translocation into the circulation, which can activate platelets and endothelial cells, contributing to thrombo-inflammatory phenomena that are increasingly recognized as part of the extra-intestinal burden of active IBD. Beyond its contribution to IBD, *Fusobacterium nucleatum* is an opportunistic pathogen with a broad disease spectrum, including periodontitis and gastrointestinal infections. Its virulence is largely mediated by RadD, FadA, and other factors that collectively promote colonization, barrier disruption, and immune activation [57,58,60,61].

## 5. *Fusobacterium nucleatum* and Colorectal Cancer Risk: Cancer Pathways and Clinical Evidence

Colorectal cancer (CRC) arises through the progressive accumulation of genetic and epigenetic alterations in the intestinal epithelium, most commonly along the adenoma–carcinoma sequence. Key pathogenic events include activation of the Wnt/β-catenin pathway, inactivation of tumor-suppressor genes such as APC and TP53, and the emergence of microsatellite instability (MSI) and CpG-island methylator phenotypes (CIMP). These molecular derangements are influenced by, and often interact with, established environmental and host-related risk factors, including advanced age, dietary patterns rich in red and processed meat, chronic intestinal inflammation, obesity, diabetes, and alterations in the gut microbiota. The latter, once considered a passive by-stander, is now recognized as an active player capable of modulating carcinogenesis and shaping the tumor microenvironment.

**Figure 1:**
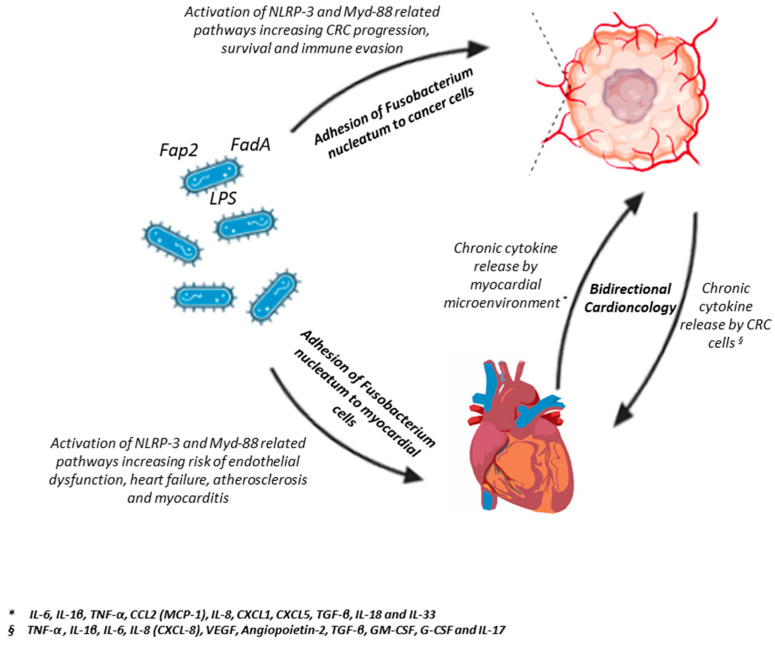
Integrated model of Fusobacterium nucleatum-driven inflammation in colorectal cancer and cardiovascular disease: a bidirectional cardio-oncologic axis. This schematic illustrates how Fusobacterium nucleatum acts as a microbial connector between colorectal cancer (CRC) and cardiovascular disease (CVD) through convergent inflammatory and signaling pathways. Via virulence factors such as FadA, Fap2, and lipopolysaccharides (LPS), *F. nucleatum* adheres to both cancer and myocardial cells, activating MyD88- and NLRP3-dependent signaling. These cascades trigger endothelial and epithelial dysfunction, promote cytokine release (IL-6, IL-1β, TNF-α), and drive CRC progression, immune evasion, atherosclerosis, myocarditis, and heart failure. The diagram further emphasizes the bidirectional cardio-oncology loop, wherein chronic cytokine release from the myocardial microenvironment (IL-6, IL-18, TNF-α, MCP-1, CXCL1, CXCL5, TGF-β, IL-33) exacerbates systemic inflammation, while tumor-derived cytokines (IL-1β, IL-6, IL-8, VEGF, Angiopoietin-2, GM-CSF, G-CSF, TGF-β, IL-17) amplify vascular injury and remodeling. Together, these reciprocal inflammatory circuits delineate a microbe-mediated cardio-oncologic axis, highlighting *F. nucleatum* as a potential shared pathogenic denominator and therapeutic target bridging oncology and cardiology. Image created by Biorender.

In recent years, *Fusobacterium nucleatum* has emerged as a compelling etiologic and prognostic factor in colorectal carcinogenesis [78,79]. Genomic and translational studies have delineated specific subspecies, most notably the Fna C2 clade, that preferentially colonize colorectal tumors and exhibit enhanced acid resistance and tumor tropism, suggesting a refined microbial driver rather than a passive passenger in oncogenesis [54]. Molecular profiling confirms that *Fusobacterium nucleatum* density is elevated in colorectal adenomas and carcinomas compared with adjacent non-neoplastic mucosa, and that its abundance correlates with adverse molecular features such as MSI, CIMP, and mutations in BRAF and TP53 [62]. Mechanistically, *Fusobacterium nucleatum* contributes to CRC development through several converging pathways. Adhesion mediated by the FadA protein to E-cadherin activates the β-catenin signaling cascade, driving oncogene expression. The lectin Fap2 binds to Gal-GalNAc moieties on tumor cells, facilitating immune evasion and remodeling of the tumor microenvironment [64]. Moreover, deep colonization of intestinal crypts promotes the emergence of cancer stem-like cells by engaging the LY6A receptor and upregulating the ribosomal protein RPS14, a factor linked to cellular hyper-proliferation [65]. Beyond direct epithelial effects, *Fusobacterium nucleatum* profoundly reshapes the immune milieu: it drives M2 polarization of tumor-associated macrophages via NF-κB activation and stimulates the secretion of pro-tumorigenic cytokines such as IL-8, CXCL1, IL-1β, IL-6, and TNF-α, thereby sustaining a chronic inflammatory and immunosuppressive microenvironment (Figure 1).

Epidemiologically, *Fusobacterium nucleatum*’s presence in stool and tumor tissue has emerged as a promising non-invasive biomarker: one study reported detection of the Fna C2 subtype in approximately 30% of patient stool samples and showed a markedly higher tumor burden in patients than in healthy controls [80]. Such specificity supports its use in risk stratification and early detection. Moreover, targeted eradication in preclinical models, through antibiotics or microbiome-directed approaches, has delayed tumor progression, while the identification of the Fna C2 subtype opens avenues for precision microbiome-mediated diagnostics and even for therapeutic delivery systems, such as “Trojan-horse” bacterial vectors capable of selectively infiltrating tumors [73]. Therefore, the current corpus of clinical and mechanistic evidence positions *F. nucleatum* not merely as a bystander but as a multifaceted architect of CRC initiation, progression, and therapeutic resistance. Its roles span epithelial signaling, regulation of stemness, immune modulation, and metabolic reprogramming, all underscored by a tangible clinical footprint, making it both a biomarker of prognostic significance and a promising target for future translational interventions in CRC prevention and treatment [81].

### Fusobacterium nucleatum and Immune Response in CRC

*Fusobacterium nucleatum* has emerged as one of the most consistently implicated microorganisms in the initiation and progression of CRC. Beyond its established role in tumor initiation through virulence factors, oncogenic microRNAs, genotoxic activity, and modulation of intestinal metabolites, the bacterium exerts a profound influence on the host immune system [82]. A central mechanism through which *Fusobacterium nucleatum* fosters tumorigenesis is the induction of chronic inflammation coupled with suppression of anti-tumor immunity (Table 2).

Upon adhering to intestinal epithelial cells, the bacterium stimulates the release of pro-inflammatory cytokines and chemokines, leading to the recruitment of inflammatory cells into the tumor microenvironment [83]. This inflammatory milieu, while initially protective, becomes a fertile ground for tumor expansion and metastatic dissemination, as it promotes epithelial proliferation, angiogenesis, and immune evasion [84]. A critical aspect of this immunomodulation is the recruitment and reprogramming of tumor-associated macrophages. *Fusobacterium nucleatum* has been shown to enhance the expression of chemokines such as CCL8, CXCL5, and leukemia inhibitory factor, which not only perpetuate leukocyte infiltration but also accelerate tumor progression [85]. This response is closely linked to activation of the TLR4/NF-κB signaling cascade in macrophages, a pathway further amplified by iron availability in the tumor microenvironment.

Elevated iron levels interfere with inhibitory phosphorylation of NF-κB, sustaining its activation and resulting in heightened secretion of tumor-promoting chemokines [86]. Clinically, this mechanism aligns with observations of iron deposition in macrophages and poorer prognoses in patients with both iron overload and high *intratumoral Fusobacterium* burden. Alongside macrophage-driven inflammation, *Fusobacterium nucleatum* profoundly influences adaptive immunity [87]. Its presence in colorectal tumors is associated with reduced densities of CD3+ T cells and selective depletion of cytotoxic CD8+ T lymphocytes, while regulatory FoxP3+ T cells accumulate, fostering an immunosuppressive niche. This skewing of the T cell compartment correlates with diminished disease-free and overall survival, particularly in stage III CRC [88] (Table 2).

Moreover, *Fusobacterium nucleatum* stimulates tumor cells directly, as infection of human colorectal cell lines induces robust secretion of pro-metastatic cytokines, including IL-8 and CXCL1, which drive epithelial migration, invasion, and metastatic potential. Such effects are strain-specific and appear more pronounced than those elicited by other Fusobacterium species, underscoring the pathogenic specialization of *Fusobacterium nucleatum* [89].

The bacterium also produces metabolites that interact with host immune pathways, further enabling immune escape and tumor persistence (Table 2). These metabolites disrupt humoral responses and contribute to immune tolerance within the tumor microenvironment, strengthening the survival advantage of transformed cells [90]. Beyond molecular mechanisms, strain-level differences within *Fusobacterium nucleatum* provide further insight into its oncogenic potential. Tumor-associated isolates are frequently classified within the subspecies animalis, which has recently been subdivided into at least two distinct clades. Of these, the C2 clade demonstrates a striking dominance in the tumor niche, suggesting that only specific phylogenetic groups of *Fusobacterium nucleatum* have the genetic and metabolic traits required to colonize neoplastic tissues effectively [91] (Table 2).

Taken together, these findings highlight *Fusobacterium nucleatum* as both a microbial driver and an immunological modulator in colorectal cancer. By integrating pro-inflammatory signaling, iron-dependent macrophage activation, T cell suppression, and metabolite-mediated immune evasion, this organism establishes a tumor microenvironment conducive to initiation, progression, and metastasis [92]. Its clade-specific enrichment within CRC underscores the need for precision microbiome profiling in risk assessment and therapeutic targeting, positioning *Fusobacterium nucleatum* as both a prognostic biomarker and a potential target for novel interventions [93].

## 6. *Fusobacterium nucleatum* and Cardiovascular Diseases: A Putative Deep Interaction

*Fusobacterium nucleatum* is predominantly recognized as a keystone pathogen in periodontal disease, yet in recent years its pathogenic role has been increasingly extended to a systemic level, particularly in cardiovascular diseases (CVD) [94]. The connection between oral microbiota and cardiovascular pathology is not merely epidemiological, but deeply mechanistic, involving bacterial dissemination, immune activation, endothelial dysfunction, and the direct remodeling of vascular structures [95]. The involvement of *Fusobacterium nucleatum* in cardiovascular diseases exemplifies the complex interplay between chronic oral infection and systemic inflammatory disorders, highlighting a shared pathophysiological continuum that bridges periodontitis, bacteremia, and atherothrombosis [96].

### 6.1. Molecular Mechanisms of Vascular Colonization and Endothelial Dysfunction

One of the principal virulence traits of *Fusobacterium nucleatum* is its ability to adhere to and invade endothelial cells through surface proteins such as FadA adhesin, which binds to vascular endothelial cadherin (VE-cadherin), destabilizing endothelial junctions and increasing vascular permeability. Once internalized, the bacterium activates pattern recognition receptors, including Toll-like receptors (TLR2 and TLR4), leading to a downstream cascade involving the NF-κB and MAPK signaling pathways [59]. This results in upregulation of pro-inflammatory cytokines such as IL-6, TNF-α, and IL-1β, along with endothelial adhesion molecules (VCAM-1, ICAM-1, E-selectin) [63]. These biochemical alterations foster leukocyte adhesion and transmigration, ultimately creating a vascular microenvironment conducive to monocyte differentiation into foam cells and subsequent atheroma development. Moreover, lipopolysaccharides derived from *Fusobacterium nucleatum* exhibit strong endotoxin activity that exacerbates oxidative stress via NADPH oxidase activation, leading to increased production of reactive oxygen species (ROS). ROS-mediated oxidation of low-density lipoprotein (oxLDL) not only accelerates foam cell formation but also destabilizes existing plaques, increasing the risk of rupture. This molecular framework provides a clear pathophysiological link between chronic periodontal colonization and systemic atherosclerotic progression [67].

### 6.2. Clinical Evidence of Systemic Dissemination

The translocation of *Fusobacterium nucleatum* from the oral cavity into the bloodstream occurs frequently in individuals with periodontal disease, particularly following episodes of gingival bleeding, tooth brushing, or dental procedures [55].

Clinical studies have documented the presence of *Fusobacterium nucleatum* DNA in carotid and coronary atherosclerotic plaques, establishing a direct microbial footprint in vascular lesions. In autopsy-based analyses, coronary plaques of patients with sudden cardiac death frequently harbor DNA fragments from periodontal pathogens, with *Fusobacterium nucleatum* among the most consistently identified species [56].

Furthermore, in patients with infective endocarditis, *Fusobacterium nucleatum* has been isolated as a causative agent, especially in cases associated with pre-existing periodontal infection. These clinical findings highlight the bacterium’s capacity to breach mucosal barriers, sustain systemic bacteremia, and seed cardiovascular tissues where it perpetuates local inflammation and tissue damage [70].

### 6.3. Preclinical Data on Immunological Crosstalk and Chronic Systemic Inflammation

The persistent exposure to *Fusobacterium nucleatum* antigens induces a state of chronic low-grade systemic inflammation, which is now considered a central feature of cardiovascular disease. Beyond localized vascular inflammation, *Fusobacterium nucleatum* triggers an adaptive immune response characterized by Th17 polarization, excessive secretion of IL-17, and activation of neutrophil extracellular traps (NETs), which further amplify vascular damage [66].

Antibody responses directed against *Fusobacterium nucleatum* surface proteins may cross-react with host antigens through molecular mimicry, potentially driving auto-immune-like phenomena that accelerate endothelial injury.

Additionally, *Fusobacterium nucleatum* produces short-chain fatty acids (SCFAs), including butyrate, acetate and propionate, primarily through amino-acid fermentation in subgingival plaques and in dysbiotic intestinal niches such as inflamed crypts, rather than by fiber fermentation as performed by commensal butyrogenic species (e.g., *Faecalibacterium prausnitzii*).

In the healthy colonic lumen, physiological butyrate concentrations, typically in the low-millimolar range (10–20 mM), are generated by fiber-fermenting commensals and serve as a critical energy source for colonocytes, supporting epithelial integrity and exerting anti-inflammatory effects.

In contrast, *Fusobacterium nucleatum*–associated butyrate is produced locally, typically at sub-millimolar levels within oral biofilms or intestinal crypts. In these microenvironments, the bacterium coexists with other pathogenic factors such as LPS, adhesins (FadA, RadD), and pro-inflammatory cytokines. SCFA exposure may influence immune-cell metabolism and histone acetylation, promoting a pro-inflammatory state, particularly where epithelial integrity is already compromised. Thus, the potential pathogenicity of SCFAs is context-dependent; it arises not from their intrinsic properties, but from their production at inappropriate sites or in combination with other virulence determinants of *Fusobacterium nucleatum*. These metabolic interactions may help explain the observed association between severe periodontitis, gut barrier dysfunction, and increased systemic inflammatory burden, which can ultimately contribute to cardiovascular risk [69] (Table 2).

*Fusobacterium nucleatum* has also been implicated in several cardiovascular conditions, reflecting the systemic impact of chronic oral infection. Notably, in the context of atherosclerosis, the bacterium promotes early endothelial activation and contributes to both plaque formation and instability. Experimental studies have shown that *Fusobacterium nucleatum* enhances lipid uptake by macrophages while impairing cholesterol efflux, leading to the generation of foam cells that form the core of atherosclerotic lesions [68]. With regard to acute coronary syndromes (ACS), the inflammatory response elicited by *Fusobacterium nucleatum*, including the release of cytokines and reactive oxygen species (ROS), can destabilize vulnerable plaques and increase the risk of rupture and thrombosis. Clinically, a higher prevalence of periodontal pathogens, *Fusobacterium nucleatum* among them, has been reported in patients presenting with myocardial infarction compared with healthy controls [11]. Although relatively rare, infective endocarditis represents another cardiovascular manifestation in which *Fusobacterium nucleatum* has been identified as a causative pathogen, particularly in individuals with poor oral hygiene or pre-existing valvular abnormalities [71]. In addition, chronic infection may contribute to heart failure, where sustained systemic inflammation and microvascular dysfunction promote myocardial remodeling. In experimental models, persistent *Fusobacterium nucleatum* exposure leads to reduced left ventricular performance and increased myocardial fibrosis, suggesting a mechanistic link between the pathogen and structural cardiac disease progression [72]. Altogether, these observations reinforce the concept that F. nucleatum–related inflammation extends beyond the oral cavity, influencing vascular integrity and myocardial health through systemic immunoinflammatory pathways.

Notably, the recognition of *Fusobacterium nucleatum* as a contributing factor in cardiovascular diseases has major therapeutic implications. Targeted periodontal therapy, including scaling, root planing, and antimicrobial treatments, has been associated with improved endothelial function and reduced systemic markers of inflammation such as C-reactive protein (CRP) [74]. Furthermore, the modulation of oral microbiota through probiotics, bacteriophage therapy, or host-directed interventions represents a promising frontier in reducing systemic inflammatory burden [75]. At a pharmacological level, inhibitors of TLR4 or NF-κB signaling may mitigate the vascular inflammatory effects of *Fusobacterium nucleatum*, although such strategies remain experimental [76]. Clinically, integrated management strategies that link dental and cardiological care are essential. Cardiovascular patients should be screened for periodontal disease, while periodontal patients should undergo cardio-vascular risk assessment. The convergence of these two disciplines emphasizes the need for a holistic view of patient health in which oral and cardiovascular systems are not treated in isolation but as intimately interconnected [77].

The relationship between *Fusobacterium nucleatum* and cardiovascular diseases represents a paradigmatic example of how a localized infection can have profound systemic consequences. Through direct endothelial invasion, activation of inflammatory cascades, oxidative stress, and immunological cross-reactivity, *Fusobacterium nucleatum* establishes itself as more than a commensal oral bacterium: it becomes a systemic pathogen with significant implications for vascular health [97]. Clinical evidence of bacterial DNA in atherosclerotic plaques, associations with myocardial infarction, and rare cases of endocarditis underscore the translational relevance of these findings. Although these findings highlight plausible mechanisms by which *Fusobacterium nucleatum* may contribute to vascular inflammation, endothelial dysfunction, and thrombo-inflammatory complications, the majority of available evidence derives from preclinical models (in vitro studies and animal experiments), with limited direct confirmation in well-characterized patient cohorts.

Future research should prioritize large, prospective longitudinal cohorts to assess microbial biomarkers in relation to cardiovascular outcomes, multicenter case–control studies with standardized detection methods, and mechanistic interventional trials that integrate microbiome profiling with vascular imaging and inflammatory endpoints. Such studies are essential to determine whether the observed associations reflect causal relationships and to define the true translational significance of *Fusobacterium nucleatum* in cardiovascular disease [98] (Table 2).

## 7. *Fusobacterium nucleatum* Strains and Disease Associations

Recent phylogenomic analyses have revealed that *Fusobacterium nucleatum* is not a single homogeneous species but a complex of genetically distinct subspecies and clades with variable virulence potential. Whole-genome and targeted gene sequencing (e.g., rpoB, 16S rRNA) have led to the recognition of four canonical subspecies, *nucleatum, polymorphum*, *vincentii*, and *fusiforme*, each characterized by specific genomic traits and ecological preferences. Subspecies nucleatum represents the reference lineage, primarily associated with oral biofilms and chronic periodontitis, while polymorphum shows greater genetic variability and has been isolated from multiple extraoral sites. *Vincentii* is more frequently linked to acute infections and abscesses, whereas fusiforme remains less studied but exhibits distinct genomic signatures. A recent high-resolution study published in Nature identified two previously unrecognized clades within *Fusobacterium nucleatum* subsp. animalis, termed Fna C1 and Fna C2. Among these, Fna C2 was found to be strongly enriched in CRC tissue and fecal samples from CRC patients, but was rarely detected in healthy individuals. Comparative genomic analyses revealed that Fna C2 harbors additional virulence factors, including fap2, cmpA, and fusolisin, and displays distinct morphology and metabolic activity compared with other subspecies. In vivo, colonization with Fna C2 significantly increased colorectal adenoma formation and altered host metabolic profiles in murine models of CRC, supporting a functional role for this clade in tumorigenesis. These findings underscore the importance of strain-level resolution in understanding *Fusobacterium nucleatum*, host interactions, and suggest that not all strains share the same pathogenic capacity. From a clinical perspective, identifying virulent lineages such as Fna C2 may inform diagnostic refinement, patient risk stratification, and targeted therapeutic approaches. Further research is warranted to determine whether Fna C2 or other high-risk clades contribute to disease processes beyond colorectal cancer, including inflammatory and cardiovascular diseases.

## 8. Pharmacological and Nutritional Approaches to Influence *Fusobacterium nucleatum* Levels

Pharmacological strategies to reduce *Fusobacterium nucleatum* colonization and systemic dissemination have primarily focused on antimicrobial therapy, modulation of host inflammatory responses, and interference with bacterial signaling pathways. Traditional periodontal treatments frequently employ systemic or locally delivered antibiotics such as metronidazole, amoxicillin, or doxycycline, which display activity against anaerobic Gram-negative bacteria, including *Fusobacterium nucleatum* [99].

However, the overuse of broad-spectrum antibiotics carries the risk of resistance development and micro-biome dysbiosis, emphasizing the importance of targeted approaches. In this regard, narrow-spectrum antimicrobial peptides (AMPs) and bacteriophage-based therapies are emerging as promising candidates to selectively eliminate *Fusobacterium nucleatum* without disrupting commensal flora [100]. Beyond direct bacterial eradication, pharmacological modulation of host responses is equally relevant. Inhibitors of Toll-like receptor 4 (TLR4) signaling and NF-κB pathway antagonists have demonstrated the capacity to attenuate *Fusobacterium nucleatum*-induced vascular inflammation in experimental models [101]. Similarly, statins and angiotensin-converting enzyme (ACE) inhibitors, widely used in cardiovascular medicine, exert pleiotropic effects on endothelial function by reducing systemic inflammation and oxidative stress, indirectly counter-acting the deleterious vascular effects of *Fusobacterium nucleatum*. Notably, statins have been shown to reduce bacterial adhesion to endothelial cells and to modulate monocyte activation, suggesting an unexpected adjuvant role in controlling microbial vascular colonization [102].

### 8.1. Nutritional Strategies and Dietary Modulation

Nutritional interventions have gained increasing attention as sustainable, long-term strategies to modulate the oral and systemic microbiome, including *Fusobacterium nucleatum* [103]. Diets rich in polyphenols, such as green tea catechins, resveratrol, and curcumin, exhibit antimicrobial properties by inhibiting bacterial adhesion, biofilm formation, and quorum sensing. In vitro studies have shown that epigallocatechin gallate (EGCG) reduces *Fusobacterium nucleatum* growth and suppresses its ability to induce IL-6 and TNF-α release from endothelial cells, thereby attenuating the pro-inflammatory cascade [104].

Omega-3 polyunsaturated fatty acids (PUFAs), particularly eicosapentaenoic acid (EPA) and docosahexaenoic acid (DHA), possess anti-inflammatory and pro-resolving properties [105]. Their incorporation into cell membranes modulates lipid raft composition and dampens TLR signaling, reducing the vascular inflammatory response to *Fusobacterium nucleatum*-derived lipopolysaccharides.

Clinical trials in periodontitis patients have demonstrated that supplementation with omega-3 fatty acids, combined with standard mechanical therapy, enhances periodontal healing and reduces systemic markers of inflammation, indirectly lowering bacterial translocation to the cardiovascular system. Prebiotic fibers and probiotics represent another nutritional avenue [106]. By selectively enriching beneficial oral and gut commensals, prebiotics reduce the ecological niches available for *Fusobacterium nucleatum*. Probiotic formulations containing *Lactobacillus reuteri* or *Bifidobacterium longum* have been shown to suppress *Fusobacterium nucleatum* colonization in the oral cavity by producing anti-microbial metabolites such as reuterin and acetate [107]. This microbiome reshaping could decrease the systemic inflammatory burden associated with chronic exposure to *Fusobacterium nucleatum*.

### 8.2. Integrative and Translational Perspectives

The integration of pharmacological and nutritional interventions may represent the most effective strategy to control *Fusobacterium nucleatum* levels and mitigate its cardiovascular impact [108]. For instance, combining periodontal antibiotic therapy with dietary polyphenols or omega-3 supplementation could simultaneously reduce bacterial load and improve vascular resilience against inflammatory insults. Moreover, host-directed pharmacological strategies, including antioxidants like N-acetylcysteine (NAC) or mitochondrial-targeted compounds, may synergize with microbiota-modulating diets to control oxidative stress and systemic immune activation triggered by *Fusobacterium nucleatum* [109]. In clinical practice, personalized approaches considering genetic predisposition, dietary habits, and oral microbiome profiling will be crucial to designing tailored interventions. The emerging field of nutrigenomics may further delineate how specific nutrients modulate host–pathogen interactions at the epigenetic and transcriptional levels, potentially opening avenues for precise modulation of *Fusobacterium nucleatum*-driven inflammation in cardiovascular patients.

## 9. Discussion

The narrative review highlights that *Fusobacterium nucleatum* acts not as a passive by-stander but as an active driver and amplifier of inflammation-driven pathology across the gut–tumor–vascular axis. In IBD, CRC, and CVD, the organism exploits conserved host interfaces, epithelial and endothelial junctions, through adhesins such as FadA and lectin-like proteins such as Fap2, disrupting barrier integrity, subverting innate and adaptive immunity, and reshaping local metabolism [110]. This unifying view of a “multi-compartmental colonization with shared inflammatory circuitry” integrates evidence from experimental and clinical studies: epithelial and endothelial engagement triggers TLR2/4–MyD88–NF-κB and MAPK cascades, inflammasome activation, ROS generation, and pro-inflammatory cytokine networks (IL-1β, IL-6, TNF-α, IL-8), while bacterial outer membrane vesicles, SCFAs and other metabolites modulate chromatin and cellular bioenergetics [111]. Clinically, this mechanistic core manifests as mucosal ulceration and relapse in IBD, immune-evasive tumor microenvironments and therapy resistance in CRC, and endothelial dysfunction with atherothrombotic events in CVD.

In IBD, *Fusobacterium nucleatum* adheres to mucins and E-cadherin via FadA, loosening adherens junctions and increasing permeability, thereby promoting translocation of bacterial products that activate lamina propria macrophages and dendritic cells, driving Th17 polarization, neutrophil recruitment and NET formation [112,113].

This inflammatory cascade is further reinforced by β-catenin activation downstream of FadA–E-cadherin engagement, linking chronic inflammation to a lower threshold for neoplastic transformation [114]. Fap2 recognition of Gal-GalNAc motifs in dysplastic epithelium and TIGIT engagement on NK/T cells explains the selective enrichment of the bacterium in CRC and its contribution to immune escape [115], in line with the NLRP3-driven, prostaglandin-rich microenvironment that promotes epithelial proliferation and oxidative DNA damage [116]. Such mechanisms translate into clinical observations: higher mucosal loads of *Fusobacterium nucleatum* correlate with endoscopic severity, steroid-refractory flares, and postoperative recurrence in ulcerative colitis [117]; these patients often display elevated fecal calprotectin, CRP, and transcriptional signatures of TLR/NF-κB and chemokine activation [118]. The bacterium’s ability to degrade mucins and disrupt tight junction proteins aligns with the increased risk of barrier failure and venous thromboembolism, with evidence that NET-driven platelet activation and tissue factor expression contribute to the pro-thrombotic state [119,120].

Moreover, in CRC, *Fusobacterium nucleatum* is consistently enriched in right-sided tumors, often in association with MSI-high and CpG island methylator phenotypes, and correlates with worse survival and higher recurrence rates. It enhances matrix metalloproteinases and EMT-related transcription factors downstream of β-catenin/ERK signaling, drives chemo-resistance via TLR4–MyD88 activation and autophagy, and modulates microRNAs involved in DNA-damage response and drug transport [121,122]. Its detection in liver metastases mirrors primary tumor colonization, suggesting dissemination with tumor cell clusters or through the portal circulation [122]. The vascular dimension links oral dysbiosis to endothelial injury. Periodontitis-related bacteremia or invasive dental procedures allow *Fusobacterium nucleatum* to seed the endothelium, disrupt VE-cadherin, activate TLR2/4 pathways, increase ROS and pro-coagulant mediators, and propagate systemic inflammation via outer membrane vesicles [123,124]. Clinical data support these mechanisms, showing associations between poor oral health and greater carotid intima-media thickness, impaired flow-mediated dilation, and atheroma instability, with *Fusobacterium nucleatum* DNA detected in atherosclerotic plaques [125].

From a translational perspective, multi-matrix detection (saliva, stool, tissue) offers windows on *Fusobacterium nucleatum* biology: salivary load reflects oral reservoir and bacteremic risk, while fecal qPCR/metagenomics correlates with mucosal burden and can augment IBD monitoring and CRC screening [126]; tumor-tissue assays may refine prognosis and guide therapy, especially where chemo-resistance pathways are active.

Standardization of sampling and analysis, together with integration of host-response readouts (e.g., fecal cytokines, NETosis markers, circulating microRNAs), may increase predictive power [127]. The sections on pharmacological and nutritional interventions fit into this framework by illustrating intervention levers that operate at three levels: (i) direct antimicrobial targeting (e.g., metronidazole, precision phages, adhesin-receptor blockade) [128]; (ii) host-directed therapies (e.g., TLR4/MyD88 or NLRP3 modulators, omega-3 PUFAs, statins/ACE-inhibitors) [129,130]; and (iii) ecological modulation (dietary polyphenols, omega-3s, prebiotic fibers, targeted probiotics) that reshape the host–microbe interface and improve biomarkers of barrier function and systemic inflammation [131,132]. Important knowledge gaps remain: distinguishing causation versus niche adaptation, resolving strain-level heterogeneity of virulence factors, disentangling confounding from concurrent therapies, and mitigating sampling/technical biases. Host genetics and environmental factors further modify colonization dynamics [133]. Addressing these gaps will require longitudinal, strain-resolved, multi-omics studies and mechanistic intervention trials with harmonized endpoints. The oral–intestinal–vascular axis unveiled here argues for integrated care pathways: routine periodontal assessment in IBD and high-risk CRC/CVD patients, gut symptom screening and cardiovascular profiling in severe periodontitis [134], and the incorporation of microbial biomarkers into monitoring and therapeutic decision-making across disciplines [135]. Future studies should test FadA–cadherin blockade for barrier and endothelial protection, Fap2–TIGIT antagonism to enhance anti-tumor immunity, and strain-specific phage therapies with mucoadhesive delivery, alongside nutrition-anchored trials targeting NETosis, epithelial lipidome and TLR signaling, and multi-matrix surveillance to pre-empt flares, treatment failure and vascular events [136,137,138].

## 10. Limitations and Controversies

Despite the growing body of evidence linking *Fusobacterium nucleatum* to intestinal inflammation, colorectal tumorigenesis, and vascular dysfunction, several uncertainties and controversies remain.

First, it is still debated whether the enrichment of *Fusobacterium nucleatum* in colorectal cancer represents a causal driver of oncogenesis or rather an opportunistic colonizer adapting to the tumor-altered microenvironment.

Tumor-associated changes such as hypoxia, altered mucin glycosylation, and necrosis may create ecological niches that favor the selective expansion of this anaerobe, thus complicating causal inference. Second, not all colorectal cancer subtypes show significant enrichment of *Fusobacterium nucleatum*; for example, some microsatellite-stable, left-sided tumors appear less frequently colonized, suggesting that host genetics, tumor molecular profiles, or local ecological factors modulate colonization patterns.

Third, most of the available data are cross-sectional, often based on single-time-point analyses of tumor or stool samples. These designs cannot determine whether microbial expansion precedes or follows neoplastic transformation or disease exacerbation. To address these gaps, longitudinal prospective studies, ideally including high-risk cohorts and serial sampling before, during, and after disease onset, are needed to clarify temporal relationships and causality. Furthermore, strain-resolved metagenomic and multi-omics approaches, coupled with functional studies and standardized sampling protocols, will be essential to distinguish pathogenic from commensal variants and to better understand microbe–host interactions.

Finally, the translational landscape remains limited by the predominance of preclinical or cross-sectional investigations. Robust longitudinal studies integrating oral, intestinal, and systemic compartments are needed to track the temporal dynamics of *Fusobacterium nucleatum* colonization and disease progression. Strain-resolved and functional metagenomic analyses will help delineate virulence diversity and clarify which genetic or metabolic traits confer pathogenic potential. Moreover, well-designed intervention trials, including antimicrobial, dietary, and periodontal therapies, should assess whether reducing *Fusobacterium nucleatum* load translates into measurable improvements in inflammatory, oncologic, or cardiovascular outcomes. Standardization of sampling methods, normalization strategies, and host biomarker integration will be essential to enable reproducibility and inter-study comparison.

These efforts will collectively bridge current gaps between experimental observations and clinical translation, defining the precise contribution of *Fusobacterium nucleatum* within the continuum linking mucosal dysbiosis, carcinogenesis, and vascular disease.

### Outstanding Research Questions

Although recent advances have illuminated many aspects of *Fusobacterium nucleatum* biology, several critical questions remain unanswered and warrant systematic investigation.

1. Strain specificity and virulence heterogeneity. Current data suggest that pathogenic potential varies markedly across *Fusobacterium nucleatum* subspecies and clades, yet strain-level determinants of virulence, such as adhesin repertoires (FadA, Fap2 variants), mobile genetic elements, and metabolic traits, remain poorly characterized. Large-scale, strain-resolved genomics and phenotypic profiling are essential to delineate pathogenic versus commensal lineages and their host tropisms.
2. Host–microbe and microbe–microbe interactions. The mechanisms by which *Fusobacterium nucleatum* interfaces with host immunity, barrier integrity, and the broader microbial community require deeper exploration. Multi-omics integration (metatranscriptomics, proteomics, and metabolomics) could unravel context-dependent interactions that modulate inflammation, tumorigenesis, and endothelial dysfunction. Defining host genetic or epigenetic susceptibilities that facilitate colonization will be pivotal for risk stratification.
3. Diagnostic and quantitative standardization. Heterogeneity in sampling matrices (saliva, stool, tissue), extraction protocols, and quantitative thresholds hampers reproducibility across studies. Standardized diagnostic pipelines, combining molecular quantification (qPCR, digital PCR, metagenomics) with host biomarkers of inflammation and immune activation, are needed to translate *Fusobacterium nucleatum* detection into clinically actionable information.
4. Therapeutic targeting and intervention studies. Despite encouraging preclinical evidence, the translational impact of modulating *Fusobacterium nucleatum* remains largely theoretical. Future trials should evaluate whether reducing bacterial burden through antimicrobial, probiotic, or dietary interventions translates into improved outcomes in inflammatory bowel disease, colorectal cancer, or cardiovascular cohorts. Mechanism-based strategies, such as blocking FadA–cadherin or Fap2–TIGIT interactions, should be explored in controlled, ethically sound frameworks.
5. Longitudinal and interventional research designs. The field urgently requires longitudinal cohort studies that track oral–intestinal–vascular colonization dynamics and intervention trials that test causality rather than association. Such designs will be crucial to move from correlative evidence to mechanistic and therapeutic validation.

Notably, addressing these outstanding questions will bridge current mechanistic insights with clinical translation, enabling precision prevention and microbiome-informed management of inflammation-driven disorders in which *Fusobacterium nucleatum* is implicated.

## 11. Conclusions

Taken together, the data support a coherent, mechanistically grounded view of *Fusobacterium nucleatum* as a trans-compartmental pathobiont that links mucosal inflammation, tumor immune escape and drug resistance, and vascular dysfunction. The organism’s conserved strategies, adhesion-mediated barrier disruption, TLR/inflammasome activation, ROS amplification, and immune checkpoint engagement, yield disease-specific phenotypes shaped by local ecology and host context. Clinically, this translates into actionable opportunities: risk stratification using standardized microbial and host biomarkers; co-management of oral health, intestinal disease, and cardiovascular risk; and interventional packages that blend precise antimicrobial pressure with host-directed and nutritional therapies. The challenge now is to convert this integrated pathophysiological model into rigorously tested, scalable care algorithms that improve outcomes across IBD, CRC, and CVD without sacrificing the integrity of the commensal microbiome that sustains human health.

## Figures and Tables

**Table 1:** Search strategy used in Medline and EMBASE.

Database	Search String
Medline	“Fusobacterium nucleatum AND colorectal cancer” OR “Fusobacterium nucleatum AND inflammatory bowel disease” OR “Fusobacterium nucleatum AND cardiovascular disease” OR “Fusobacterium nucleatum AND immune modulation” OR “oral microbiota AND systemic diseases”
EMBASE	“Fusobacterium nucleatum AND colorectal cancer” OR “Fusobacterium nucleatum AND IBD” OR “Fusobacterium nucleatum AND cardiovascular disease” OR “Fusobacterium nucleatum AND microbiota”

**Table 2:** Comparative overview of molecular mechanisms and clinical evidence linking *Fusobacterium nucleatum* to inflammatory bowel disease (IBD), colorectal cancer (CRC), and cardiovascular disease (CVD).

Mechanism	Molecular Pathway	Pathogenic Effects in IBD	Pathogenic Effects in CRC	Pathogenic Effects in CVD	Preclinical Evidences	Clinical Evidences
**Mucosal colonization and persistence**	Isolation from intestinal biopsies; subspecies tropism (*Fna C2* clade in CRC)	Persistent presence in IBD mucosa, driving local inflammation and barrier disruption	Selective enrichment in right-sided CRC; enhanced tumor colonization and acid resistance	Bacteremia and vascular tissue seeding following oral dissemination	In vitro and in vivo models confirm mucosal adhesion and invasion [51,52,53,54]	Frequent detection in IBD biopsies and CRC tissues; *F. nucleatum* DNA in vascular plaques [51,52,55,56]
**Adhesion and epithelial/endothelial invasion**	Adhesins FadA, RadD binding to E-/VE-cadherin	Disrupts epithelial junctions, increases permeability	Activates β-catenin signaling → oncogene transcription and epithelial proliferation	Alters endothelial junctions and vascular permeability	Cellular invasion assays; β-catenin pathway activation [53,57,58,59]	Elevated FadA expression in CRC and CVD lesions [57,58,59]
**Biofilm formation and interspecies aggregation**	Outer membrane proteins RadD, Fap2	Supports microbial persistence and mucosal colonization	Enhances biofilm stability in tumor niches, promoting immune evasion	Facilitates oral–vascular dissemination via stable multispecies biofilms	Demonstrated interbacterial co-aggregation in vitro [60]	Identified in oral biofilms from IBD and periodontitis patients [60]
**Host signaling and inflammatory activation**	FadA–E-cadherin → β-catenin; TLR2/4 → NF-κB, MAPK	Induces IL-6, TNF-α, IL-1β release, amplifying mucosal inflammation	Promotes oncogenic signaling, proliferation, and cytokine production	Upregulates VCAM-1, ICAM-1, and E-selectin → leukocyte adhesion, foam-cell formation	Functional models confirm cytokine cascades and NF-κB activation [61,62,63]	Elevated inflammatory mediators in affected tissues [62,63]
**Immune evasion and immunomodulation**	Fap2–Gal-GalNAc binding; TIGIT inhibition; macrophage polarization	Modulates local immune response and delays mucosal healing	Suppresses NK and T-cell cytotoxicity; promotes M2 macrophage phenotype	Induces Th17 polarization, IL-17 production, and NET formation	Immune-cell assays and murine CRC/CVD models [64,65,66]	Fap2 detected in tumor and vascular tissues; Th17 signature in patients [64,66]
**Stemness and crypt colonization**	LY6A receptor activation → RPS14 upregulation	Not reported	Induces cancer stem-like cell phenotype, sustaining proliferation	Not reported	Observed in colonic crypts of experimental models [65]	Association with aggressive CRC subtypes [62,64,65]
**Endotoxin activity and oxidative stress**	LPS → NADPH oxidase activation	Contributes to epithelial injury and oxidative stress	Sustains tumor microenvironmental inflammation	Generates ROS, oxidized LDL, and plaque destabilization	Pro-oxidant and pro atherogenic effects in vitro [67]	Strong correlation with plaque inflammation and instability [67,68]
**Metabolic and epigenetic modulation**	Local SCFAs (butyrate, acetate, propionate)	Alters immune-cell metabolism and histone acetylation in crypts	May modulate tumor cell signaling under dysbiotic conditions	Promotes vascular inflammation when co-occurring with LPS and barrier injury	SCFA exposure experiments in cell and animal models [69]	Observed link between SCFA imbalance and inflammatory burden [69]
**Systemic dissemination and direct infection**	Bacteremia, translocation from oral niches	Extraintestinal flares linked to oral inflammation	Hematogenous spread to extraintestinal tumors (breast, pancreas)	Infective endocarditis; bacterial DNA in heart valves and atheromas	Animal and in vitro infection models [55,56,70,71]	Detection of *F. nucleatum* DNA in endocarditis and vascular lesions [55,56,70,71]
**Chronic inflammation and tissue remodeling**	Cytokine cascade (IL-6, TNF-α, IL-8, CXCL1)	Drives recurrent flares and mucosal ulceration	Reinforces tumor growth and invasion	Promotes endothelial damage and myocardial fibrosis	Induced chronic inflammation in experimental models [65,72]	Correlation with disease severity and cardiac dysfunction [65,72]
**Therapeutic implications**	Targeted eradication; periodontal therapy; TLR4/NF-κB inhibition	Reduces local inflammation, improves barrier repair	Attenuates tumor growth; potential microbial “Trojan horse” vectors	Improves endothelial function and lowers CRP; experimental inhibitors in development	Preclinical antibiotic and microbiota-modulating trials [73,74,75,76]	Clinical periodontal interventions improve vascular outcomes [74,75,76,77]

## Data Availability

Not applicable.
